# Parents’ Knowledge and Awareness About the Importance of Primary Teeth and Space Maintainers in Saudi Arabia: A Cross-Sectional Study

**DOI:** 10.7759/cureus.61836

**Published:** 2024-06-06

**Authors:** Alaa K Redwan, Hesham A Alhazmi, Sharifah A Alharthi, Jomana J Alharbi

**Affiliations:** 1 Preventive Dentistry, Umm Al-Qura University, Makkah, SAU; 2 General Dentistry, Umm Al-Qura University, Makkah, SAU

**Keywords:** oral hygiene, parents’ knowledge, oral health, primary teeth, space maintainer

## Abstract

Background: Primary teeth are important for eating, speaking, and esthetics, as well as for guiding the eruption and maintaining the space for the permanent teeth. The best space maintainers (SM) for permanent dentition are the natural primary teeth. The aim of this study was to assess parents’ knowledge and awareness of the importance of primary teeth and SM in Saudi Arabia.

Method: This cross-sectional study invited parents who were living in Saudi Arabia via different social media platforms to complete a validated questionnaire. The data collected included demographic characteristics, questions about parents’ knowledge of their children’s oral hygiene, and the importance of primary teeth, as well as questions about knowledge of SM.

Results: A total of 392 participants completed the questionnaire. Dental problems and pain were the most common reasons for visiting the pediatric dentist (n=260; 66.3%). With respect to the age at which the first primary tooth erupted, more than half of the parents (n=270; 68.9%) knew the correct age. Only 43.3% (n=168) of the parents had received any information about SM and only 39.5% (n=155) of them knew their purpose.

Conclusion: Most parents agree that treating primary teeth is important for their children’s oral health and development. However, most are unaware of an SM’s purpose. We recommend that dentists discuss SM’s importance with parents actively after extracting their child’s teeth. This will help increase parents’ awareness and understanding of SM and encourage them to be more informed about this dental treatment option.

## Introduction

Oral health is part of one’s health overall and is recognized as a crucial component of the quality of life (QoL) [1]. Primary teeth are important for eating, speaking, and esthetics, as well as for guiding the eruption and maintaining the space for the permanent teeth [2-4]. The majority of the population believes that the permanent teeth will replace the primary teeth and for that reason, the primary teeth are rarely given the attention needed for treatment by parents [2-4]. On the one hand, parents who have appropriate attitudes and knowledge about oral health are more likely to have a positive influence on their child’s dental health. On the other, parents’ lack of understanding and awareness of oral health will influence their children’s attitudes and behavior adversely in the future [3,5]. Furthermore, most parents still take their children to the dentist for corrective rather than preventive care [4]. Therefore, it is important to further assess and improve parental attitudes and knowledge regarding primary teeth and their significance [3]. It should be noted that the pediatric dentist must instruct the patient and their parents properly on the way to take care of the primary and permanent teeth [6].

Dental caries is a major worldwide health problem [7]. The American Dental Association defines it as a “… biofilm-mediated, sugar-driven, multifactorial, and dynamic illness that leads to the phasic demineralization and remineralization of dental hard tissues” [8]. According to a previous systematic review, the prevalence of dental caries in primary teeth was 46.2% worldwide [9]. Moreover, a recent cross-sectional study published in 2023 that measured dental caries’ prevalence and severity in school children in Saudi Arabia showed that dental caries’ prevalence overall was 65.6%, with 72.1% of primary teeth and 61.7% of permanent teeth affected [10]. It has been determined that poor oral hygiene and lack of awareness are major behavioral risk factors for dental caries that may lead to the premature loss of primary teeth [7,11]. Another factor that may contribute to increasing caries risk in children is family income, as it has been determined that children from low-income families have higher caries prevalence [3].

The best space maintainers (SM) for permanent dentition are the natural primary teeth [4]. If primary tooth loss is unavoidable, it may lead to loss of space and results in crowding, impaction, and super-eruption of the opposing teeth, which could lead to malocclusion and lifelong consequences in the permanent dentition [4]. To avoid these consequences, the space created by the early loss of primary teeth must be preserved by using an SM appliance [2]. This is used primarily to maintain the arch length after the premature loss of primary teeth to prevent unwanted teeth drifting and migration [12]. However, very few studies have been conducted in Saudi Arabia that measured parents’ knowledge and awareness of the primary teeth’s importance and SM’s effectiveness [12-14]. Thus, this study assessed parents’ knowledge and awareness of the primary teeth’s importance and SM’s effectiveness in Saudi Arabia.

## Materials and methods

Ethical consideration

Ethical approval for this study was obtained from the institutional review board (IRB) of Umm Al-Qura University and no study activities began until IRB approval was obtained (Approval No. HAPO-02-K-012-2023-03-1507). Written consent was obtained from all participants in the study, and the data were collected anonymously.

Study design

This cross-sectional study targeted the population of parents residing in Saudi Arabia who were recruited from social media platforms. The questionnaire was distributed through WhatsApp, Telegram, and Snapchat. 

Inclusion and exclusion criteria

Parents who live in Saudi Arabia and are able to read Arabic were included in the study. However, parents who did not give consent to participate in the study were excluded.

Sample size calculation and sampling technique

A prevalence of 40% of awareness about SM among parents, a confidence interval of 95%, and an alpha level of 5% were used to calculate the minimum sample size required. The estimated sample size was 380 participants [4]. Convenience sampling was used to recruit parents and the questionnaire was formulated initially in English and translated into Arabic later. The questionnaire was validated by pilot testing the survey with 12 participants before the data collection process began. The survey was delivered to participants via Google form in an online link and could be completed in less than 10 minutes.

The questionnaire

The questionnaire consisted of 29 questions and was formulated with the guidance of previous studies with some minor modifications [2-4,6,12-13,15]. The questionnaire consisted of four sections. The first included the study’s purpose and the consent form. The second included questions about demographic characteristics. The third contained questions about parents’ knowledge of their children’s oral hygiene and the importance of primary teeth, and the fourth included questions about parents’ knowledge of SM.

Statistical analysis

The data were collected, tabulated, and analyzed statistically using SPSS v. 20. Descriptive statistics were performed to present data on the demographic distribution. The analysis focused on examining the parents’ knowledge of their children’s primary teeth and the knowledge of SM. Chi-square tests were conducted to assess the association between the demographic variables and receiving information about SM (Yes=1; No=0). The significance level was set at a level of p-value <0.05.

## Results

Data collection took place from March 2023 to June 2023. A total of 392 met the criteria and were able to complete it. The participants’ demographic characteristics are presented in Table 1. Among them, 42.9% (n=168) had more than three children, and 75% (n=294) were females. Approximately half of the participants (n=199; 50.8%) were between the ages of 26 and 40. The majority of the participants (n=370; 94.4%) were Saudi and lived in the Western region (n=320; 81.63%). More than half of the participants (n=261; 66.6%) held a bachelor’s degree, and a similar proportion (n=207; 52.8%) were employed. A total of 32.1% (n=126) of respondents reported a monthly family income that ranged from 5000 to 10000 SAR. There was no statistically significant difference across different demographic groups and whether the parents received information about SM (p-value >0.05).

**Table 1:** Demographic characteristics of the study population. n: number of participants; SAR: Saudi Riyal

Question	Answer	Frequency n (%)
Number of children	1	61 (15.6%)
	2	90 (23.0%)
	3	73 (18.6%)
	More than 3	168 (42.9%)
Gender	Male	98 (25.0%)
	Female	294 (75.0%)
Age	<25	32 (8.2%)
	26-40	199 (50.8%)
	Older than 40	161 (41.1%)
Nationality	Saudi	370 (94.4%)
	Non-Saudi	22 (5.6%)
Region	Western region	320 (81.63%)
	Other	72 (18.63%)
Education	High school or less	70 (17.9%)
	Bachelor's degree	261 (66.6%)
	Master's/PhD degree	61 (15.6%)
Employment status	Student	10 (2.6%)
	Employee	207 (52.8%)
	Unemployed	131 (33.4%)
	Retire	44 (11.2%)
Monthly income	Less than 5000 SAR	112 (28.6%)
	5000-10000 SAR	126 (32.1%)
	10000-20000 SAR	113 (28.8%)
	More than 20000 SAR	41 (10.5%)

The results of the parents’ knowledge about their children’s oral hygiene and the primary teeth’s importance are presented in Table 2. Among the parents, 66.58% (n=261) took their child to a pediatric dentist for the first time after the child was two years old. While 89.5% (n=351) of the parents were aware of the importance of dental visits, 66.3% (n=260) of them reported that dental problems and pain were the most common reasons for visiting the pediatric dentist. Most of the parents (n=385; 98.2%) agreed that their child’s primary teeth should be brushed and 91.7% (n=355) of them helped their child brush their teeth and 75.5% (n=296) used fluoridated products. Only 15.8% (n=62) of the parents reported that they use dental floss to clean their child’s teeth. With respect to the age at which the first primary tooth erupted, more than half (n=270; 68.9%) of the parents knew the correct age. However, only 34.9% (n=137) of them knew the correct total number of primary teeth, although most of them (n=357; 91.1%) acknowledged the importance of treating primary teeth.

**Table 2:** Knowledge of parents about their children’s oral hygiene and the importance of primary teeth. n: number of participants

Question	Answer	Frequency n (%)
Age when my kid first visited the pediatric dentist	1 year or less	66 (16.84%)
	1-2 years old	65 (16.6%)
	2 years or more	261 (66.58%)
Is it important to visit the pediatric dentist?	Yes	351 (89.5%)
	No	41 (10.5%)
What was the reason for visiting the pediatric dentist?	Routine checkup	106 (27%)
	Dental problem/pain	260 (66.3%)
	Did not visit at all	26 (6.6%)
Is it important to treat primary teeth?	Yes	(91.1%) 357
	No	35 (8.9%)
Do you agree about brushing your child's primary teeth?	Agree	385 (98.2%)
	Disagree	7 (1.8%)
If you agree, do you help your child with tooth brushing?	Yes	355 (91.7%)
	No	32 (8.3%)
Do you use fluoride products to clean your baby's teeth?	Yes	296 (75.5%)
	No	96 (24.5%)
Do you use dental floss to clean your child's teeth?	Yes	62 (15.8%)
	No	330 (84.2%)
What is the age of the first primary teeth eruption?	Less than 6 months	84 (21.4%)
	6-12 months	270 (68.9%)
	More than 1 year	38 (9.7%)
What is the total number of primary teeth?	Correct answer	137 (34.9%)
	Wrong answer	255 (65.05%)

Table 3 shows the questions about SM. Of all of the parents, 57.6% (n= 266) reported experiencing their child missing primary teeth. The reasons for the missing teeth were as follows: 48.29% (n=141) because of physiological factors, 32.19% (n=94) because of caries, and 17.46% (n=51) attributable to trauma. Among the parents, 27.5% (n=108) of them reported that their dentists were the main source of information about SM, while 56.7% (n=220) had not heard about them at all. Furthermore, only 39.5% (n=155) of parents knew SM’s purpose, while 60.5% (n=237) were unaware of their purpose or had inaccurate information about their purpose. Only 15.1% (n=59) of the parents reported that their child received space maintainer treatment.

**Table 3:** Parental experiences with early loss of primary teeth and the use of SM in children. n: number of participants; SM, space maintainers

Question	Answer	Frequency n (%)
Do you have personal experience of a child missing primary teeth?	Yes	226 (57.6%)
	No	166 (42.3%)
What was the reason for the loss?	Physiological	141 (48.29%)
	Caries	94 (32.19%)
	Trauma	51 (17.46%)
	Others	6 (2.10%)
How did you receive information about SM?	Dentist	108 (27.5%)
	Friends or family member	19 (4.9%)
	Social media	41 (10.6%)
	Didn’t hear about it before	220 (56.7%)
What is the purpose of SM?	Correct answer	155 (39.5%)
	Wrong answer	58 (14.80%)
	I don't know	179 (45.7%)
Have you ever received space maintainer treatment for your child?	Yes	59 (15.1%)
	No	333 (84.9%)

## Discussion

This cross-sectional study aimed to assess parents’ knowledge and awareness of the primary teeth’s importance and SM’s effectiveness in Saudi Arabia. Around 66.58% (n=261) of the parents in our sample took their child to the dentist at the age of two years or more. In fact, the first dental visit should range from as soon as the first primary tooth erupts and no later than 12 months of age according to the American Academy of Pediatric Dentistry’s recommendations [16]. A previous study that Manohar and Mani conducted reported that only 10% of the parents knew the correct age for the first dental visit [15]. However, in our study, only 16.84% took their children to the dentist at age one or less.

Our survey showed that 89.5% (n=351) of parents knew about the importance of visiting pediatric dentists, and 91.1% (n=357) of them agreed that it is important to treat primary teeth. This finding was similar to a previous study that assessed the prevalence of premature loss of primary teeth among children in Dammam City, Saudi Arabia. They found that more than 60% of parents agreed that primary teeth are as important as permanent teeth and more than 75% of parents take their children to the dentist every six months [11]. However, 66.3% (n=260) of the parents in our study took their child to the dentist only if they complained of pain, similar to that in bin mhna et al.’s study, which reported that dental pain was the cause of 46.1% of pediatric dentist visits [13]. Furthermore, in a study that fatmah AlMotawah et al. conducted, they found that 54.2% of parents take their children to the dentist only in case of emergency [14]. 

In our study, 98.2% (n=385) of parents agreed that their children’s primary teeth should be brushed, and 91.7% (n=355) helped their children while they brushed their teeth. This was similar to the result of a cross-sectional study that Nassar et al. performed, which measured the knowledge of parents regarding early childhood caries prevention of preschool children in the western region of Saudi Arabia and showed that 63% of the participating parents did not believe that their child could brush their own teeth alone effectively [17]. In contrast to parents’ agreement that their children’s teeth should be brushed, our study showed that 84.2% (n=330) of them did not use dental floss to clean their children’s teeth. This was reported as well in a previous study by bin mhna et al., in which 77.7% of the participants did not use dental floss to clean their children’s teeth [13]. Several questions were asked in our study to evaluate parents’ general knowledge about the primary teeth’s importance. We found that more than half (n=270; 68.9%) of them chose the correct age at which the first tooth erupts, while 65.05% (n=255) were unaware of the total number of primary teeth. 

Our survey revealed that 57.6% (n=226) of parents experienced their child’s primary tooth loss, but only 15.1% (n=59) of them received an SM. This finding might indicate their lack of knowledge of space maintenance. In a previous study published in 2020, 56.3% of parents had personal experience with their child missing primary teeth and over 94% did not receive SM treatment for their children [4]. Furthermore, in another study published in 2022, the authors reported that 90.4% of the participants had never received an SM [14]. A study was conducted to determine the parents’ level of knowledge of SM’s importance for their children and reported that 76% of participating parents had not heard about SM, which also did not differ statistically significantly when compared to their education level [14]. 

In this study, more than half of parents (n=220; 56.7%) had not received any information about SM. These findings were similar to those in studies that Alduraihim et al. and Ali et al. performed, where they found that 82.1% and 73.7% of participants, respectively, lacked information about SM [4,6]. In our study, 27.5% (n=108) of parents reported that the dentist was their source of information regarding SM, which is considered a relatively low percentage. Similarly, another study found that only 19% of participants received information from their dentist [6]. With respect to SM’s purpose, only 39.5% (n=155) of the parents in our study knew their correct purpose. This is consistent with Linjawi’s research that noted that the level of Saudi Arabian parents’ knowledge of SM’s importance and use was very low [12]. We suggest that the general dentist should have good knowledge about the importance of primary teeth and SM to make parents aware of SM and the way to manage early tooth loss. Furthermore, initiating parental awareness campaigns about the importance of primary teeth and the use of SM might improve the parent's oral health knowledge.

As with all cross-sectional studies, ours was particularly susceptible to recall bias, as the answers were self-reported, and the convenience sample we used to recruit participants may have contributed to sampling bias because the researcher chose the sample based on unequal probability. Furthermore, this research engaged a limited number of fathers, which can be considered a study limitation as it makes it impossible to compare fathers' and mothers’ answers. In addition, the majority of the study’s participants were from the western region with only a small number of participants from other regions; thus, the sample may not represent the entire population. Finally, conducting an online survey on social media cannot be representative of all Saudi Arabian parents. Future studies should seek to involve more fathers and individuals from different regions. Nonetheless, this study conducted an assessment that may encourage general and pediatric dentists to foster awareness among parents in Saudi Arabia about the importance of primary teeth and SM use. For future studies, other study designs might be considered to overcome the limitations of the cross-sectional studies.

## Conclusions

In our study, most parents agree that treating primary teeth is important for their children’s oral health and development. However, most are unaware of an SM’s purpose. We recommend that dentists discuss SM’s importance with parents actively after they extract their child’s teeth. This will help increase parents’ awareness and understanding of SM and encourage them to be more informed about this dental treatment option.

## References

[1] Spanemberg JC, Cardoso JA, Slob EM, López-López J (2019). Quality of life related to oral health and its impact in adults. J Stomatol Oral Maxillofac Surg.

[2] Ramakrishnan M, Banu S, Ningthoujam S, Samuel VA (2019). Evaluation of knowledge and attitude of parents about the importance of maintaining primary dentition - a cross-sectional study. J Family Med Prim Care.

[3] Vittoba Setty J, Srinivasan I (2016). Knowledge and awareness of primary teeth and their importance among parents in Bengaluru city, India. Int J Clin Pediatr Dent.

[4] Alduraihim HS, Alsulami SR, Alotaibi SZ, El-Patal MA, Gowdar IM, Chandrappa PN (2020). Assessment of Saudi parent's awareness towards space maintainers at Alkharj city: a cross-sectional study. J Family Med Prim Care.

[5] Hamasha AA, Rasheed SJ, Aldosari MM, Rajion Z (2019). Parents knowledge and awareness of their children’s oral health in Riyadh, Saudi Arabia. Open Dent J.

[6] Ali A, Hebbal M, Aldakheel N, Al Ghamdi N, Eldwakhly E (2022). Assessment of parental knowledge towards space maintainer as an essential intervention after premature extraction of primary teeth. Healthcare (Basel).

[7] Mubaraki S, AlOlyan R, AlBrekeit J, AlFouzan S, Abosharkh M, AlSaeri N, Baseer MA (2022). Prevalence of caries in first permanent molar among children in Saudi Arabia: a retrospective study. Eur Rev Med Pharmacol Sci.

[8] (2023). Caries Risk Assessment and Management. https://www.ada.org/resources/ada-library/oral-health-topics/caries-risk-assessment-and-management.

[9] Kazeminia M, Abdi A, Shohaimi S, Jalali R, Vaisi-Raygani A, Salari N, Mohammadi M (2020). Dental caries in primary and permanent teeth in children's worldwide, 1995 to 2019: a systematic review and meta-analysis. Head Face Med.

[10] Orfali SM, Alrumikhan AS, Assal NA, Alrusayes AM, Natto ZS (2023). Prevalence and severity of dental caries in school children in Saudi Arabia: a nationwide cross-sectional study. Saudi Dent J.

[11] AlMeedani LA, Al-Ghanim HZ, Al-Sahwan NG, AlMeedani SA (2020). Prevalence of premature loss of primary teeth among children in Dammam city and parents’ awareness toward space maintainers. Saudi J Oral Sci.

[12] Linjawi AI, Alajlan SA, Bahammam HA, Alabbadi AM, Bahammam MA (2016). Space maintainers: Knowledge and awareness among Saudi adult population. J Int Oral Health.

[13] Nasser G, Hamdi S, Alsadoon M (2022). Primary teeth importance and dental care of children among mothers in Saudi Arabia: knowledge and awareness assessment. Med Sci.

[14] AlMotawah F, Al Thani N, Al Qudrah A, Al Shabi M, Jammoul Y, Al Alwan M (2022). Parent’s awareness of the importance of space maintainers in Riyadh; a cross-sectional study. Arch Pharm Pract.

[15] Manohar J, Mani G (2017). Knowledge and attitude of parents regarding children’s primary teeth & their willingness for treatment. J Pharm Sci.

[16] American Academy on Pediatric Dentistry; American Academy of Pediatrics (2009). Policy on early childhood caries (ECC): classifications, consequences, and preventive strategies. Pediatr Dent.

[17] Nassar AA, Fatani BA, Almobarak OT, Alotaibi SI, Alhazmi RA, Marghalani AA (2022). Knowledge, attitude, and behavior of parents regarding early childhood caries prevention of preschool children in Western Region of Saudi Arabia: a cross-sectional study. Dent J (Basel).

